# Bulk elastic properties of chicken embryos during somitogenesis

**DOI:** 10.1186/1475-925X-9-19

**Published:** 2010-03-30

**Authors:** Ubirajara Agero, James A Glazier, Michael Hosek

**Affiliations:** 1 The Biocomplexity Institute and Department of Physics, Swain Hall West 157, 727 E. 3rd Street Bloomington, IN 47405-7105 USA; 2 Departamento de Fisica, Universidade Federal de Minas Gerais, Caixa Postal 702, Belo Horizonte, CEP 31.270-901, Brazil

## Abstract

We present measurements of the bulk Young's moduli of early chick embryos at Hamburger-Hamilton stage 10. Using a micropipette probe with a force constant *k *~0.025 N/m, we applied a known force in the plane of the embryo in the anterior-posterior direction and imaged the resulting tissue displacements. We used a two-dimensional finite-element simulation method to model the embryo as four concentric elliptical elastic regions with dimensions matching the embryo's morphology. By correlating the measured tissue displacements to the displacements calculated from the in-plane force and the model, we obtained the approximate short time linear-elastic Young's moduli: 2.4 ± 0.1 kPa for the midline structures (notocord, neural tube, and somites), 1.3 ± 0.1 kPa for the intermediate nearly acellular region between the somites and *area pellucida*, 2.1 ± 0.1 kPa for the *area pellucida*, and 11.9 ± 0.8 kPa for the *area opaca*.

## Background

Somitogenesis is a key early stage of animal development, during which the initially continuous presomitic mesoderm (*PSM*) on each side of the neural crest, segments into the periodic somites that later give rise to the vertebrae and associated structures. Both avian and mammalian somitogenesis require large-scale reorganization of PSM cells and associated extracellular matrix (*ECM*), as cells from the PSM condense to form the somites [1,2]. Under brightfield illumination, somites appear as compact, rounded tissues adjacent to the neural crest and notocord, with both medial and lateral gap regions free of cells. The visible embryonic morphology suggests that the mechanical properties of Hamburger-Hamilton (*HH*) stage 6-14 embryos [3] are spatially complex.

**Figure 1:**
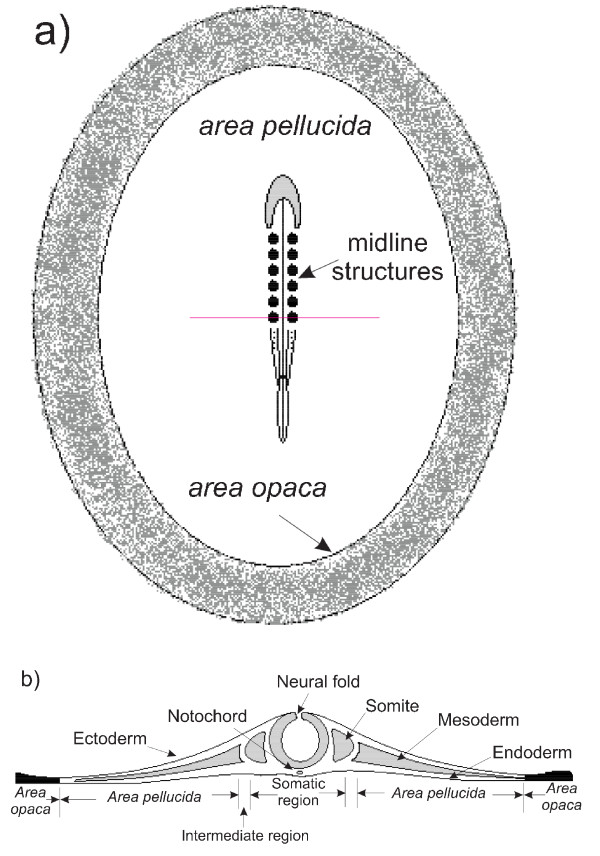
**a) Schematic dorsal view showing the key regions of a HH 12 chick embryo**. b) Transverse cross section of chick embryo at the anterior-posterior position of the last somite indicated by the horizontal line in a.

Figure 1 shows a dorsal view of the embryo and a transverse cross section at the anterior-posterior position of the somites. Informal mechanical manipulations of embryos indicate that the embryonic tissue is relatively stiff and that the cohesive ECM prevents the PSM and somites from easily separating from their surroundings. This structural complexity suggests that local elasticity plays a major role in describing the structural rearrangements at this stage of development.

Despite extensive efforts to identify the genes and signals involved in somitogenesis [1,4,5], mechanical studies are lacking. Perhaps, because of the absence of biomechanical data, most models of somitogenesis [6,7] focus on its biochemical aspects. However, the mechanical properties of tissues are important for both somitogenesis and development more generally, since cells generate and respond to mechanical forces as they rearrange to define the shapes and sizes of embryonic structures [8-10]. For example, cultured myocytes growing on different substrates can sense the mechanical properties of their environment and change their morphology in response to the substrate stiffness [11]. Models of the wing imaginal discs of *drosophila *indicate that mechanical forces can determine the final size of tissues [12,13]. Compressive forces applied to osteoblasts can induce gene expression of ECM proteins [14]. In a situation with many parallels to somitogenesis in chick, mechanical boundary conditions determine the movement and reshaping of tissues during body-axis elongation in *Xenopus *[15]. Indeed, based on his studies of gastrulation, Keller and collaborators [16] have proposed that morphogenesis is fundamentally biomechanical, inspiring a number of biomechanical models of early development [17-19]. A complete understanding of tissues must delineate how the interactions of the genome, the cytoskeleton, and cell-cell and cell-ECM interactions scale up to bulk tissue properties. For example, recently Zhou *et al*. [20] have found, somewhat surprisingly, that the cell actin cytoskeleton plays a major role in early embryo stiffness, relative to the fibronection component of the ECM.

Since studies of the biomechanics of development must begin with quantitative measurements of embryonic-tissue mechanical properties, numerous researchers have studied tissue mechanics in specific cases. Forgacs and others [21] have used compression apparatus to measure the viscoelastic behavior of spherical cell aggregates reconstituted from embryonic tissues extracted from limb bud, liver, heart and retina. Moore and coworkers, also using a compression method, have measured how the elastic modulus changes in time for explants of the involuting marginal zone from *Xenopus laevis *[22]. Recently Wiebe and Brodland, using cantilever-applied forces, elongated tissues and measured the stress of extracted parts of embryonic epithelia from *Axolotl *[23]. Zamir and Tabler have applied a microindentation method to measure the elastic properties and residual stress in early embryonic chick heart [24]. Murayama and colleagues have also used indentation of the *area pellucida *of bovine *ovum *to measure its Young's modulus [25].

However, no measurements are available for HH 6-14 stage chick embryos. This paper presents a simple experimental technique to measure the bulk short time linear-elastic Young's moduli for avian embryos.

To measure the elastic properties of chick embryos, we developed an instrument to apply a controlled force at a specific point in the embryo. We used a three-axis micromanipulator mounted on an inverted microscope to position a glass micropipette at a desired position in the embryo. By moving the pipette horizontally, we applied an in-plane force to the embryo. The bending angle of the calibrated micropipette gave the applied force. We then measured the resulting tissue displacements. Because calculating the Young's moduli from the applied stress (force) and observed displacement (stress), is formally *ill posed*, (*i.e*., many possible moduli could give the same displacement fields), we need additional structural information about the embryo to calculate the Young's moduli. We therefore measured key morphological parameters of the embryo which we believe correspond to the primary domains of different moduli, and constructed a simple finite-element model of the tissues based on these measurements. Fitting the observed displacements for the known applied force to the predicted displacements then gave the specific values of the Young's moduli for the different embryonic regions. This method should allow simple determination of the elastic properties of other quasi-two-dimensional tissues. Because we are interested in tissue elasticity, our measurements focused on short-term stress-strain relations. This could be extended to study long term viscoelastic effects [20] using longer duration and displacement.

## Results

### Displacement measurements

**Figure 2:**
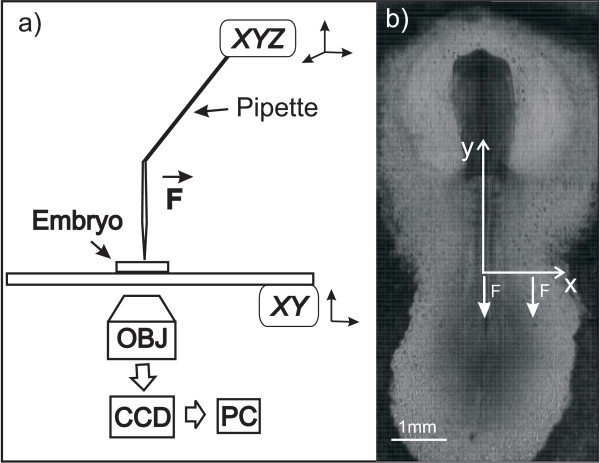
**a) Experimental schematic**. The embryo sits on the *xy *stage of an inverted microscope. A programmed displacement of a glass micropipette attached to an *xyz *controller generates a force in the plane of the embryo. We capture the image using a CCD camera attached to the microscope. b) Image of an embryo showing the axis definitions and the points of force application. During the experiments we applied AP force either at the center or at the side of the embryo, in the middle of the *area pellucida*, as indicated by the arrows. Even though we use a hollow pipette, there is no suction applied to the tissue; it used as a force probe in the horizontal *xy *plane, and is inserted 50 μm into the tissue.

We measured displacement fields for anterior-posterior (*AP*) forces applied at two positions in the embryo, as seen in Figure 2. We call the anterior-posterior axis of the embryo the *y *axis and the position of the pipette *y*_0_, *x*_0_. In the first case, we applied force *F*_*M *_along the mediolateral midline of the embryo, between the last-formed pair of somites, usually the tenth pair to form. In the second case, we applied force *F*_*AP *_in the *area pellucida*, at a point lateral to the tenth somites and half-way between the somites and the *area opaca*, (in the mediolateral (*ML*) direction and aligned with the AP direction.) The points of force application are defined by the sizes of the regions in the embryo, which vary about 10% from embryo to embryo, as can be seen in Table 1.

**Table 1:** Finite-element model morphological parameters.

Subregion	Ellipse Axes	Thickness
midline	*S*_*b *_2.2 ± 0.3 mm	84 ± 13 μm
	*S*_*a *_0.18 ± 0.02 mm	
	*N *= 10	*N *= 5

*area pellucida*	*AP*_*b *_2.8 ± 0.2 mm	56 ± 11 μm
	*AP*_*a *_1.15 ± 0.1 mm	*N *= 5

*area opaca*	*R*_*AO *_5 mm	76 ± 22 μm
		*N *= 5

The sizes were measured directly from the microscope images of multiple embryos.

The outer margin of the *area pellucida *is not very well defined, but this error is less than that due to the variations between embryos.

While visualizing the embryo in the microscope, we lowered the micropipette, concurrently making small movements in the plane of the embryo to indicate when the micropipette touched the embryo's surface. We then lowered the pipette an additional 50 μm into the embryo and waited a few minutes until we saw no flow in the fluid surrounding the embryo. The pipette easily punched through the embryonic tissue, and we checked that the tissue was not displaced from its position before penetration. Even though we use a hollow pipette, there is no suction applied to the tissue. We then moved the base of the pipette a controlled distance while filming the embryo at a rate of five frames per second. Typically, we repeated each displacement four times. The actual applied force was in the range 100 nN to 430 nN for different experimental measurements.

The embryo stopped moving immediately after pipette movement; we observed no viscoelastic effects at our resolution of 0.2 to 5 seconds, although plastic deformations could certainly occur over longer times (many minutes to hours). Each movement produced the same displacements within 10%. We do not investigate such long-term relaxation in this paper, though our technique can certainly do so.

**Figure 3:**
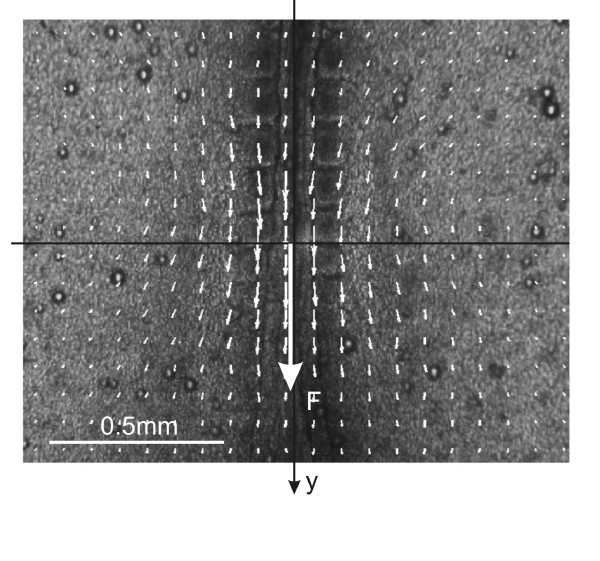
**Typical PIV calculation of the displacement field for a force applied along the midline**. In this example, we applied a force *F *= 190 nN in the caudal direction at the AP position of the last-formed somite pair (large arrow). Small arrows represent the local average displacements for square subregions of the embryo. The displacement scale is magnified compared to the image scale; the displacements in the center are on the order of 5 μm. The actual data set is a lattice of 40 by 32 displacement vectors; for clarity only a 20 by 16 sublattice is displayed. The images are 640 by 512 pixels.

We selected an image with no force applied as a reference and one frame with force applied to measure the displacement field in the embryo due to the force. We checked that the embryo returned to its original configuration when we removed the applied force, verifying that images taken before and after force application were the same, which indicated that no creep occurred for the small forces and displacement times used. To derive tissue displacements from the images, we used public-domain Particle Image Velocimetry (*PIV*) algorithms http://urapiv.wordpress.com/. PIV algorithms compare sub-regions of image pairs and use cross-correlation to determine their local relative displacements [26]. PIV is widely used in fluid dynamics, materials science, and for measuring the forces single cells exert on substrates [26-29]. The optical texture of the embryo had enough contrast that we did not need to use dyes or tracker particles to obtain clear displacement patterns. Figure 3 shows a representative image and the calculated displacement field.

### 2D finite-element model of the chick embryo

**Figure 4:**
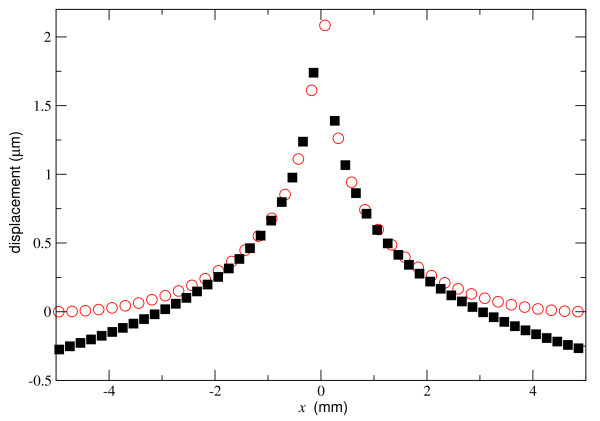
**Comparison of the calculated *y *displacement along the force direction for two-dimensional elasticity theory (filled squares) and a finite-element model (open circles) for a point-force displacement**. The theoretical displacement goes to negative infinity at large distances. The parameters for the calculations and model are: fixed radius is 5 mm, Young modulus is 2 kPa, Poisson's ratio is 0.3, thickness is 50 μm and force is 200 nN.

The early chick embryo is nearly planar, at most a hundred micrometers in thickness and several millimeters in diameter, suggesting that an analytic two-dimensional elastic theory [30,31] could be adequate to model the embryo. However, the two-dimensional solution for an elastic sheet with in-plane displacement has a logarithmic behavior which makes it a poor match to an embryo with a rigid boundary, as Figure 4 shows.

Consequently, we developed a computational simulation of the embryo's mechanical behavior using finite-element methods. To account for tissue-thickness changes in the embryo, we developed a two-and-a-half-dimensional (two-dimensional with a simplified treatment of thickness effects) planar-stress model using measured morphological parameters and tissue thicknesses over chosen subregions. The embryos were mounted on a circular paper ring of radius 5 mm, which fixed the outer boundary in both experiment and model. Corresponding to the visually apparent structure of the embryo, we defined either three or four ellipses representing the regions of the embryo, each with a specific thickness and Young's modulus.

**Figure 5:**
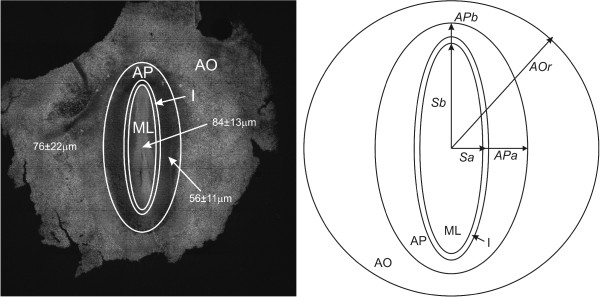
**a) Representative image of a chick embryo showing the average thickness and boundaries of the model subregions, constructed by averaging image planes obtained using two-photon microscopy**. Subregions AO: *area opaca*; AP: *area pellucida*; ML: midline structures; and I: Intermediate region. b) Structural model of the embryo (not to scale) showing the notation for the model subregions. Refer to Table 1 for the average sizes and thicknesses derived from microscope images. The paper ring, which serves as a fixed boundary condition, has radius *AO*_*r*_.

In our three-region model we represented the boundary between the *area pellucida *and the *area opaca *as an ellipse with major and minor axes extracted from the actual embryo dimensions. We represented the embryo's midline structures, containing the neural groove, head, somites and presomitic mesoderm by another ellipse, again using averaged experimental measurements, as illustrated in Figure 5. Table 1 gives the sizes of these regions. The induced displacements the embryos developed across the region between the somites and the *area pellucida *indicated that this region is quite soft. Hence we also employed a four-region finite-element model which included an intermediate region between the midline structures and the *area pellucida*. The intermediate region was an ellipse concentric with the midline ellipse, and 50 μm greater in both principle radii. The thickness of the intermediate region was the same as for the area *pellucida*.

We assumed that each subregion had a uniform thickness, Young's modulus, and linear elastic response, then used the different thicknesses to rescale the other parameters. We set the Poisson's Ratio to 0.3 in our model, but varying it over the range from 0.1 to 0.4 changed the calculated displacement field by less than 10%, which is less than our displacement-measurement error. This relative insensitivity to Poisson's ratio is the reason we could not determine it using our technique. The subregion thickness rescales the stress/strain relation (the measured *xy *displacement is proportional to the *xy *stress divided by the product of the Young's modulus and the thickness). We neglected some aspects of visible morphology including the attachments between the somites, the notocord, and neural crest, *etc*., and any slippage between the tissues above and below the ectoderm. Because the tissues below the ectoderm seem relatively soft when manipulated and appear to move with the same displacements as the ectoderm, we feel that this approximation introduced a negligible additional error. However, we plan to investigate the spacial variation of stiffness in three dimensions in future experiments.

**Figure 6:**
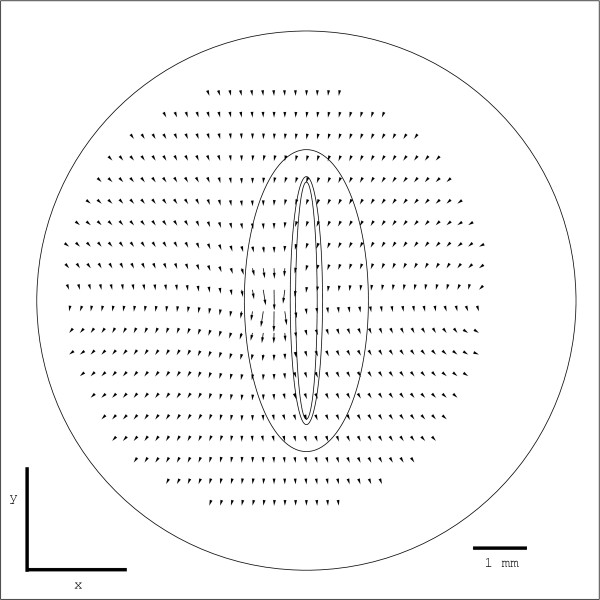
**Displacement field calculated from a four-region finite-element model; the force *F*_*AP *_is applied at the middle of the *area pellucida***. The ellipse axes correspond to the values in Table 1. The longest arrow represents a displacement of approximately 7 μm. This image corresponds to the appropriate numerical solution (after about 100 iterations) of Figure 9.

Given hypothetical values for the Young's moduli of the subregions and the known applied force, we could numerically calculate a displacement field. By iteratively adjusting the hypothetical Young's moduli, we could match the model's displacement field to the experimental displacement field. To further reduce the model degeneracy, we optimized the radii to minimize the RMS error between the model displacement fields and both experimental displacement fields, the one with the force applied along the midline and the one with the force applied at the midpoint of the *area pellucida*. Figure 6 shows a typical simulated model displacement field.

## Discussion

In the linear-elastic approximation, the magnitude of the stress field scales directly with the applied force, so we could normalize the ensemble of displacement fields by the applied force and sum them to create an averaged displacement field. Averaging reduces the noise in the individual displacement fields, which is large because the pixel resolution of the images is about 1.5 μm and the maximum displacement is about 5 μm (Figure 3). Because of the sensitivity of our calculations to small variations in the displacement field, we obtained much more accurate Young's moduli using this averaging. Since the variations in embryo subregion sizes were small, about 10% in Table 1, we assumed the subregions were identical for all embryos. Table 2 shows the more accurate Young's moduli calculated by averaging before inversion. This treatment of the data is analogous to the image-averaging methods used in astrophotography.

**Table 2:** Young's moduli obtained from the averaged experimental displacement field.

Subregion	Young's modulus (kPa)
midline	2.4 ± 0.1

intermediate	1.3 ± 0.1

*area pellucida*	2.1 ± 0.1

*area opaca*	11.9 ± 0.8

Young's moduli obtained from the averaged experimental displacement field using the four-domain finite-element model. The RMS residual between the model displacement field and the averaged displacement field was 0.17 μm.

**Figure 7:**
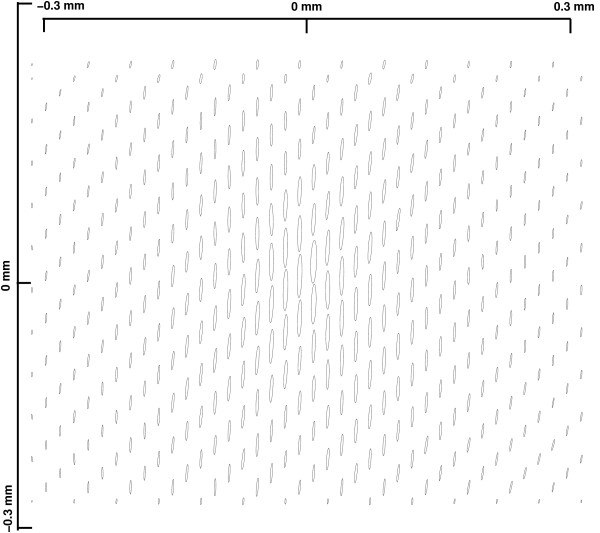
**Averaged experimental displacement field for an AP force applied along the midline**. The major ellipse axis represents the magnitude and direction of the displacement, and the minor axis represents the standard deviation. To facilitate visualization, the ellipses' axes are upscaled by a factor of ten compared to the image. We show one third of the PIV domains in the lattice.

**Figure 8:**
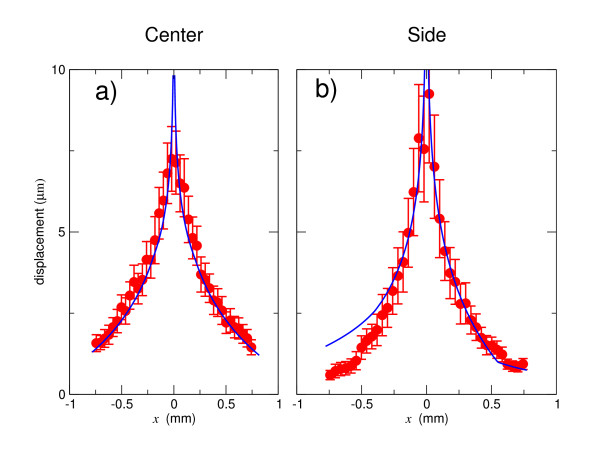
**The *y *component of the averaged experimental displacement field (points) compared with the calculated displacement from the four-region finite-element model with the same applied force (lines), calculated for a locus of points along the mediolateral *x *axis (at *y *= 0)**. a) Force applied along the embryo's midline. b) Force applied at the midpoint of the *area pellucida*.

Figure 7 demonstrates the noise-reducing effect of averaging. The narrow ellipses show that the errors in the calculated displacement were of the order of 0.2 μm. A typical individual displacement field derived from a PIV calculation fluctuated much more in magnitude and direction. Figure 8 shows that the four-region model reproduced the observed averaged displacements. The lattice of displacements is 40 × 32 elements (Figure 3); the total error between the model and this set of displacements is 500 μm^2^, or an average RMS error of about 0.6 μm per lattice site.

To compare our results with literature data, we needed to range rather widely, since we could not find results for chick embryos. A micro-tactile sensor technique [25] shows that bovine *ovum *has a Young's modulus of 25 ± 8 kPa. Force measurements by Green [32] on the *zona pellucida *of fertilized hamster eggs imply a Young's modulus of 25 kPa, as deduced from their values of force (10-30 nN) and area (2.8 μm^2^), and measured change of 40% in the thickness of the *zona pellucida*. Amphibian embryos seem to have a very small Young's modulus. Moore and colleagues [22] measured Young's moduli of 3 - 30 Pa for the involuting marginal zone of *Xenopus laevis*, and Wiebe and Brodland [23] measured a Young's modulus of 20 Pa for embryonic epithelia, but these measurements are for embryonic stages much earlier than ours in an organism with a spherical blastula. Young chick embryos (before HH stage 4) are very fragile and fall apart very easily, suggesting their Young's moduli are much smaller than in later stages. Jain and collaborators [33] measured Young's moduli of 20 kPa to 34 kPa for a composite material made from collagen sponge (a soft tissue used in wound healing) seeded with fibroblasts. One of the most revealing studies [20] shows that the bulk modulus of *Xenopus *increases from approximately 10 Pa to 90 Pa between stages 11 and 21.

These large tissue-to-tissue, species-to-species, and stage-to-stage variations indicate the need to measure Young's moduli at the correct location and embryonic stage and in the correct species rather than attempting to extrapolate from other species or earlier or later time points in the same species.

## Conclusions

While our three- and four-domain models had similar residuals, our unbiassed fit of the four-domain model calculated a smaller Young's modulus for the intermediate region than for the surrounding regions, as we observed in our experiments. We thus believe our four-subregion results to be more reliable.

In future work we will correct for embryo-to-embryo variations in subregion size, develop more realistic finite-element models of region shapes based on more detailed morphology studies, and study both asymmetry and viscoelastic effects (frequency dependence) of the Young's moduli in the embryo. By applying displacements in multiple directions and at multiple sites, we hope to be able to extract the Poisson's ratios as well as more accurate Young's moduli. We also plan to study biopolymer gels, which will serve as reference models for complex living tissues.

Because the major contributor to variations in the modeled Young's moduli for individual embryos is the noise in the measured displacement field, achieving greater accuracy in imaging will be one of our primary goals.

## Materials and methods

### Chicken embryos

We incubated eggs from White Leghorn Chickens (Purdue University-Animal Sciences) at 37°C for about 34 hours to obtain HH stage 10 embryos. We extracted the embryos with a filter-paper ring following the first steps of the protocol described in [34], put them ventral side up in a cell-culture dish (Corning Incorporated, 35 × 10 mm) containing a drop of Ringer's saline solution to avoid direct contact with the dish. We then transferred the dish to the microscope stage for measurements, which lasted about 20 minutes. We made our measurements at room temperature ~21°C. Chick embryos are remarkably resistant to temperature variations and do not require a CO_2_-enhanced atmosphere. However, to check that the period at room temperature had not affected the viability of the embryos, after measurement we maintained randomly-selected embryos according to the culture protocol at 37°C for an additional 12 hours and verified that their growth was indistinguishable from that of embryos directly transferred to culture dishes and not subjected to measurements.

### Experimental apparatus

We imaged the chick embryos on an inverted microscope (Olympus IMT-II) using a 4× objective attached to a USB CMOS camera (Mightex, MCE-B013-U). Figure 2a shows the experimental setup, including a bent micropipette attached to an *xyz *micromanipulator (Sutter Instrument Co., MPC-325). We mounted the micromanipulator on the microscope base and attached a glass micropipette to the micromanipulator grip, so we could move the sample on the microscope stage and the micromanipulator/micropipette independently.

We made the micropipettes from glass capillary tubes (Sutter Instrument Co., internal diameter 0.78 mm; external diameter 1.0 mm) using a micropipette puller (Sutter Instrument Co. P-87), configuring the puller to produce long micropipette tips, with an external diameter of about 6 μm. If necessary, we removed the end of the micropipette tip with forceps to leave a 6 μm diameter tip. In each case, we checked that the tip was a long cylinder with a diameter increasing by no more than 1 μm at 1000 μm from the tip. We then heated the capillary tube with a small Bunsen burner and bent the micropipette near its midpoint, taking care not to damage the tip of the micropipette. A bent capillary was necessary to avoid degrading the microscope illumination during the experiment. We attached the micropipette to the *xyz *micromanipulator and lowered it into the embryo, penetrating 50 ± 2 μm. Moving the micropipette applied force at the penetration position in the direction of tip displacement. In each experiment, we moved the micropipette base ± 20 μm in the AP direction in the embryo plane, displacing regions of the embryo away from the penetration point on the order of a few microns. Figure 2b shows the axis definitions and points where we applied the force. In each experiment, we moved the base of the pipette caudally from *y*_0 _to a position *y*_0 _+ 20 μm, paused approximately two seconds, moved to *y*_0_, paused approximately five seconds, moved rostrally to *y*_0 _- 20 μm, paused approximately two seconds, then moved back to *y*_0_. For position descriptions, see Figure 2.

### Micropipette calibration

We needed to calibrate each micropipette after each experiment to determine the forces we applied to the embryo. We modeled the micropipette tip as a cylinder and measured the drag force as a function of its velocity in silicon oil (Dow Corning 200) with a viscosity = 0.934 Pa·s. We inserted the tip of the pipette into the oil, to the same depth as in the embryo, 50 ± 2 μm, and filmed the tip and oil while displacing the microscope stage with different velocities. We measured the oil velocity by monitoring the displacement of impurities (small pieces of glass) placed on the oil surface. While the velocity stayed constant only for brief intervals, these intervals were long enough for the micropipette tip to reach terminal velocity in the oil as indicated by a constant displacement of the pipette tip.

The drag force for a cylinder moving with its axis perpendicular to a fluid is [35]:

where **F **is the drag force, *L *the cylinder length, *R *the cylinder radius, **v **the fluid velocity, and *η *the viscosity of the fluid. In our case *L *= 50 μm, and *R *= 3 μm. The calibration curve yields the force constant of the micropipette; typically *k *= |**F**|/*x *= 0.0275 ± 0.00075 N/m. This error was much smaller than the errors in the measured displacement fields.

### Measurements of embryo morphology

To construct a finite-element model of the embryo required several basic morphological parameters. We measured the sizes of different regions of the embryo directly (see Figure 1b) using bright-field images acquired on the inverted microscope we used in our experiments. To measure the thickness of the subregions we used a multiphoton laser-scanning confocal microscope (Leica SP2) in two-photon mode to examine fixed embryos.

To fix the embryos, we initially followed the same protocol as for our mechanical measurements, then soaked the embryos for 2 hours in a solution of 0.4% v/v of glutaraldehyde in Ringers solution, with 10 mM MOPS (morpholinepropanesulfonic acid, Sigma) pH 7.1 at 4°C. We then raised the temperature to 20°C for 20 minutes and washed the tissue and stored it in Ringers solution at 4°C. For fluorescent labeling, we cut the embryos from their paper rings with a scalpel and soaked them in BAB (borate buffer, 50 mM NaBO_3 _with 50 mM NaCl, pH 9.0) for 2 hours, then washed in 10 mM glycerol/BAB and labeled overnight in 1 mg/10 mL fluorescein-5-thiosemicarbazide/BAB (Molecular Probes F121) at 4°C. We obtained two-photon confocal *z*-stack images with 5.6 μm slice spacing for five embryos. Since the dye labeled all embryonic tissues, we could determine the thickness of the embryo as a function of AP and ML position with a resolution of 6 μm. To measure the thickness of the subregions, we choose about 6 different positions in each subregion and followed the *z*-stacks to identify the points where the tissue appeared and disappeared. Figure 5 shows a representative image of an embryo and the associated thickness measurements and errors obtained for five embryos with two or three measurements in each region.

### Numerical aspects of the modeling

As described earlier, we estimated the Young's moduli by optimizing the match between the measured displacements **u**_*m*_(**X**) with the a displacement field **u**_*c*_(**G**), calculated from our model. **G **is a finite element mesh generated by 2D Delaunay triangulation, with approximately 3000 nodes; **X **is a 40 by 32 rectilinear grid of points, as in Figure 3. We interpolate **u**_*c*_(**G**) to obtain **u**_*c*_(**X**), *i.e*. the calculated displacement at positions **X**. We define the error as Σ_*i*_|**u**_*c*_(**X**_*i*_) - **u**_*m*_(**X**_*i*_)|^2^. In pseudo-code, the optimization is as follows

   choose initial values E1..E4 for the Young's moduli

   while (error > error_max) {

      error = Σ_*i*_|**u**_*c*_(**X**_*i*_) - **u**_*m*_(**X**_*i*_)|^2^.

      generate new E1..E4 guess with least-squares fitting routine.

   }

Fixed parameters for the calculation are: region geometry (Figure 6 and Table 1), region thickness (Table 1), and the known external force. Region geometry and thickness are derived from the embryo's morphology. The varied model parameters are the four (or three) Young's moduli, listed in Table 2.

Additional constraints are obtained by simultaneously calculating **u**_*cM *_from a force **F**_*M *_applied at the midline and **u**_*cAP *_from a force **F**_*AP *_applied at the *area pellucida*. These are two separate calculations on two different meshes, but the optimized Young's moduli values are, of course, common to both. The error is the sum of the two individual errors Σ_*i*_|**u**_*cM*_(**X**_*i*_) - **u**_*mM*_(**X**_*i*_)|^2 ^+ Σ_*i*_|**u**_*cAP*_(**X**_*i*_) - **u**_*mAP*_(**X**_*i*_)|^2^.

**Figure 9:**
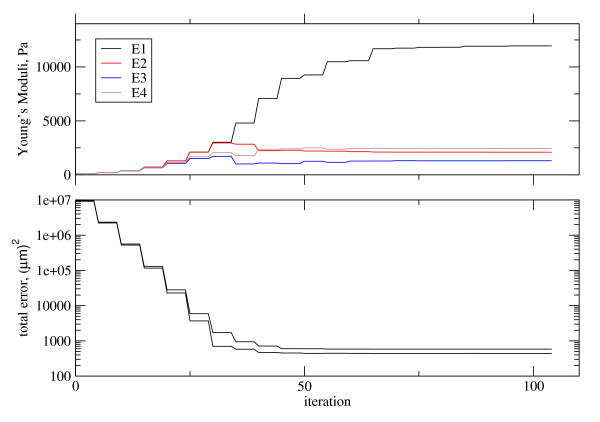
**Here two data sets are being modeled by two corresponding finite-element calculations, both sharing common Young's moduli**. In (a), the bad initial guess of 100 Pa for each Young's moduli is seen to converge to the values of Table 2. In (b), the two error functions are seen to be rapidly minimized by the least-squares optimization routine. One finite-element calculation corresponds to a force applied at midline (*F*_*M*_) and the other corresponds to a force applied at *area pellucida *(*F*_*AP*_). *E*_1_, *E*_2_, *E*_3_, and *E*_4 _correspond to the Young's moduli in the *area opeca*, *area pellucida*, intermediate region, and midline region, respectively.

We used a Comsol Multiphysics http://www.comsol.com structural mechanics subroutine to calculate **u**_*c*_(**G**), and used the matlab http://www.mathworks.com routine lsqcurvefit (based on a trusted-region reflective search algorithm) to seek the optimal Young's moduli. As seen in Figure 9, given a very bad initial guess for the Young's moduli, the optimization algorithm is able to converge within 100 iterations. The code is available as supplementary online information.

We find that the major contributor to variations in the modeled Young's moduli for individual embryos (Table 3) is noise in **u**_*m*_. Individual embryo measurements show a signal-to-noise ratio of approximately one for displacement, whereas the averaged ensemble displayed a relative variance of approximately 0.10. This latter value is qualitatively demonstrated in Figure 8.

**Table 3:** Calculated Young's moduli from individual displacement fields.

Subregion	Young's modulus (kPa)
midline	2.9 ± 2.4

intermediate	1.2 ± 0.9

*area pellucida*	2.6 ± 2.0

*area opaca*	11.6 ± 6.7

We rejected the ten experimental displacement fields where the model had residual errors in the calculated displacement greater than 0.5 μm, so *N *= 39.

See Additional file 1 for specific examples of the software and data used in the numerical calculations.

## Competing interests

The authors declare that they have no competing interests.

## Authors' contributions

UA conceived and executed the experiments, and performed the calculations. JAG provided guidance and edited the text. MKH implemented the embryo-morphology measurements and edited the text. Each author has read and approves the final manuscript.

## Supplementary Material

Additional file 1 This is a compressed html file with specific examples of the software and data used in the numerical calculations. Click here for file
