# Supplementary material for: Bulk elastic properties of chicken embryos during somitogenesis
Source: Biomed Eng Online. 2010 Mar 30;9:19. doi: 10.1186/1475-925X-9-19 (PMC3212891; doi:10.1186/1475-925X-9-19)
Supplement: Additional file 1 — This is a compressed html file with specific examples of the software and data used in the numerical calculations. [file 1475-925X-9-19-S1.ZIP › YM.zip!/index.html]

 Comsol/Matlab code used in finding optimal Young's moduli 


### Comsol/Matlab code and data used in finding optimal Young's moduli

---

Here is the code and data files used to generate Figure~9.
Comsol Multiphysics 3.5 and matlab 7.4.0 (2007a) were used.

---

To use this, first start Comsol. Within Comsol,
"File : Client/server/MATLAB : connect to MATLAB".
A matlab window opens up. In the matlab window,
go to the proper directory and execute file fit4Davg.m.

The top routine is 
fit4Davg.m .
It reads in the data files ..\averages\sidemedia.txt and
..\averages\centermedia.txt, which are the averaged measured displacement
on a lattice. It uses matlab routine
lsqcurvefit
with the error function
CenterSide4Dmkh.m 
to optimize the
Young's moduli; this routine uses
center4Dmkh.m 
and
Side4Dmkh.m 

YM.zip 
is a zip file with the code and data files.

---
